# Ingestion-activated CGRP neurons control learning but not satiety

**DOI:** 10.1101/2025.10.08.681275

**Published:** 2025-10-09

**Authors:** Brooke C. Jarvie, Anagh Ravi, Rhea Nanavati, Longhui Qiu, Truong Ly, Jun Y. Oh, Olivia K. Barnhill, Zachary A. Knight

**Affiliations:** 1 Department of Physiology, University of California, San Francisco, San Francisco, CA 94158, USA; 2 Kavli Center for Fundamental Neuroscience, University of California, San Francisco, San Francisco, CA 94158, USA; 3 Neuroscience Graduate Program, University of California, San Francisco, San Francisco, CA 94158, USA; 4 Howard Hughes Medical Institute, University of California, San Francisco, San Francisco, CA 94158, USA

## Abstract

Calcitonin Gene-Related Peptide (CGRP) neurons in the parabrachial nucleus are critical for sickness and malaise but have also been proposed to control non-aversive (rewarding) satiation. How one cell type can coordinate these opposing processes has not been explained. Here we reinvestigate the function of these cells using single-cell imaging and optical manipulations. Contrary to current models, we show that CGRP neurons do not track cumulative food consumption, and their activity is not necessary for meal termination or satiety. Instead, we identify two distinct populations of CGRP cells, one of which responds rapidly to appetitive signals during ingestion and the other of which responds slowly to aversive visceral cues. Surprisingly, the ingestion-activated CGRP neurons are important for learning about post-ingestive effects but do not control ongoing food consumption. This reveals two populations of CGRP neurons that are sequentially engaged during, and responsible for, the distinct stages of post-ingestive, aversive learning.

## Introduction

The decision to stop eating can arise from either satiety, which is rewarding, or sickness, which is aversive. How these two internal states are differentially represented in the brain is a longstanding and unresolved question in neuroscience.

Calcitonin Gene-Related Peptide (CGRP) neurons in the parabrachial nucleus (PBN) are a cell type that sits at the interface between sickness and satiety. Stimulation of CGRP neurons robustly inhibits feeding, and these neurons are by activated an array of signals that induce nausea or visceral malaise^1–3^. Moreover, CGRP neuron stimulation is itself aversive, and activation of these cells is necessary and sufficient for learning about many aversive cues^4–10^. This has led to the hypothesis that CGRP neurons function as a “general alarm” that signals danger, especially as it relates to internal state^11^.

Nevertheless, a separate line of research has suggested that CGRP neurons also play an important role in physiologic (non-aversive) satiation and meal termination. This is supported by the finding that silencing of CGRP neurons delays meal termination and can also attenuate behavioral responses to injection of some gut hormones that promote satiety^3^. These observations raise the question of how the same population of cells can represent both nausea, which is aversive, and satiety, which is rewarding.

Relatively little is known about the single-cell dynamics of CGRP neurons during ingestion^12^. One study reported that CGRP neurons were homogeneously activated by both aversive and nutritive signals, albeit on different timescales^12^. Here, we have reinvestigated how CGRP neurons are regulated in different behavioral contexts in order understand how they control both satiety and sickness.

## Results

### A subset of CGRP neurons is rapidly activated in a manner time-locked to ingestion

**Figure 1,:**
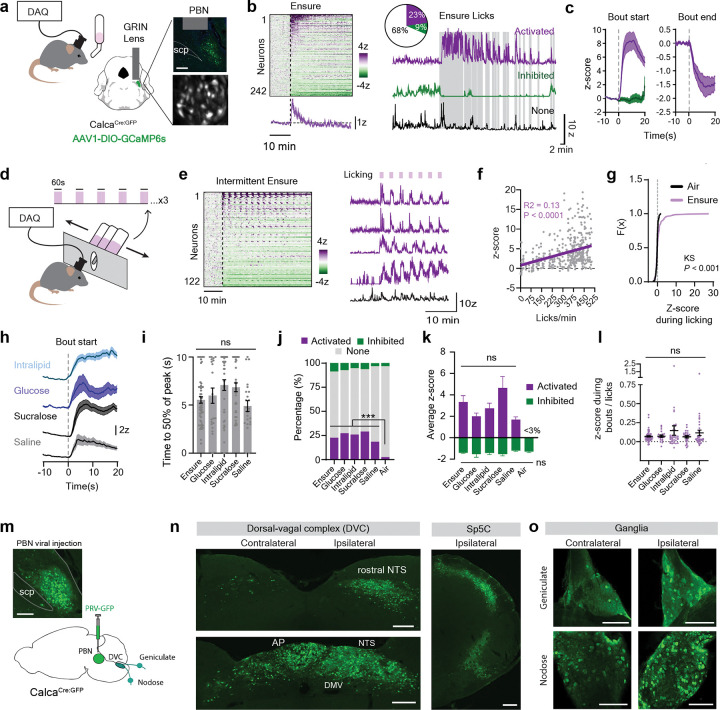
A subset of CGRP neurons rapidly tracks ingestion. **a**, Single-cell imaging of CGRP neurons in the parabrachial nucleus (PBN) during self-paced licking. Right, Representative lens placement and GCaMP6s expression in CGRP neurons and view through the GRIN lens. scp, superior cerebellar peduncle. **b**, Left, heatmap and average CGRP neuron population response to Ensure consumption. Right, quanitfication and example of types of neural responses to Ensure. Vertical gray bars represent licks. **c**, Peri-stimulus time histogram (PSTH) of CGRP neuron responses relative to the first and last lick in a bout. **d**, Schematic of mice being given intermittent access to Ensure on the Davis Rig. **e**, Heatmap of neural responses to intermittent licking and example neural traces. **f**, Correlation between licking and mean z-scored response. **g**, CDF of CGRP neuron responses when licking air vs Ensure. **h**, PSTH of activated neurons in response to licking for various solutions. **i**, Time to 50% of peak response following first lick in a bout. **j**, Quantification of neural responses. **k**, Average z-score during the first 10 minutes of solution access of activated and inhibited neurons. **l**, Average neural responses during bouts, normalized to the number of licks. **m**, Injection of the multi-synaptic retrograde pseudorabies virus-GFP (PRV-GFP), and a representative image of viral expression in the PBN. **n**, Expression of PRV-GFP in cells upstream of CGRP neurons in the dorsal vagal complex and the ocaudal spinal trigeminal nuclues (Sp5C) 3 days after injection of PRV-GFP. **o**, PRV-GFP expression in the geniculate and nodose ganglia. Data are represented as mean ± sem. AP, area postrema. NTS, nucleus of the solitary tract. DMV, dorsal motor vagus. Scale bars, 200 μm. ns means not significant, *** P < .001,

To investigate the regulation of PBN-CGRP neurons during food consumption , we targeted expression of GCaMP6s to these cells by injecting a Cre-dependent AAV into the PBN of *Calca*^Cre:GFP^ mice^1^ and in the same surgery installed a GRIN lens for microendoscopic imaging (Fig. 1a, Supplemental Fig. 1a). We then gave hungry mice access to the complete liquid diet Ensure and performed single-cell calcium imaging during self-paced ingestion (30 minutes). We found that 23% of CGRP neurons were rapidly activated during Ensure consumption in a manner that was time-locked to licking (mean bout activation: 3.3 ± 0.6 z, Fig. 1b–c). This lick-triggered activation began with the first lick, ramped over several seconds (50% of peak activation at 5.5 ± 0.3 sec) and then gradually declined when the lick bout ended (50% decrease after 6.7 ± 0.4 sec, Fig. 1c). Similar time-locked, lick-triggered responses were observed when mice were given intermittent access to Ensure in one minute trials (Fig. 1 d–e), and the magnitude of this response scaled with the size of the lick bout (R^2^ = 0.130, *P* < 0.0001, Fig. 1f). These responses were consistent at the single-cell level, with activated neurons responding in 79 ± 3% of licking trials.

We next sought to define the sensory signals that activate CGRP neurons during ingestion. First, we noted that CGRP neuron activation was not simply due to the mechanical act of licking, because less than 3% of neurons responded when mice performed “air licking” at an empty bottle (KS test of CGRP neuron response to licking air vs. Ensure, *P* = 0.0002, Fig. 1g). On the other hand, this effect was not macronutrient specific, because consumption of a pure fat solution (intralipid) or a pure sugar solution (glucose) caused time-locked activation of CGRP neurons (Fig. 1h–i, Supplemental Fig. 1b) that was similar to Ensure in both the percentage of responsive cells (Fig. 1j) and the strength of their activation (mean z-score: Ensure 3.3 ± 0.6, intralipid 2.7±0.5, glucose 2.0±0.3, comparison of all conditions *P* = 0.55, Fig. 1k). We also observed strong CGRP neuron activation during consumption of the non-nutritive sweetener sucralose and ingestion of saline (a mildly salty solution, Fig. 1i–k). Mice sampled all solutions provided, and despite some differences in volume consumed (Supplemental Fig. 1c), normalizing the neural activation during ingestion to the number of licks did not reveal any differences between any solutions tested (Fig. 1l). We did observe a small subset of CGRP neurons that were tonically inhibited during our imaging sessions, but these responses did not correlate with ingestion (Fig. 1j–k, Supplemental Fig. 1b). Taken together, these data indicate that a subset of CGRP neurons are rapidly activated during ingestion by a signal that depends on the volume consumed but is independent of nutrient identity or caloric value.

### CGRP neurons receive extensive input from pregastric sensory pathways

The time-locked activation of CGRP neurons during ingestion suggests that these neurons may be modulated by sensory input from the mouth or esophagus^13–16^. To identify possible sources of this pregastric feedback, we performed polysynaptic retrograde tracing to visualize the inputs to CGRP neurons. We did this by injecting a Cre-dependent pseudorabies virus expressing GFP (PRV-Introvert-GFP, Fig. 1m)^17^ into the PBN of *Calca*^CreGFP^ mice and examining the spread of virus at timepoints 2–4 days after infection (Fig. 1n–o, Supplemental Fig. 2a–c).

On day 2, there was extensive labeling of neurons in regions associated with visceral malaise (area postrema)^18–20^, taste (rostral NTS)^21–23^, and feedback from the esophagus and lower GI tract (medial and caudal NTS^24,25^, Supplemental Fig. 2b)^8,26–29^. On days 3 and 4, we observed the appearance of GFP expression in peripheral sensory neurons. This included labelling in the geniculate ganglia, which contains the cell bodies of the neurons that carry gustatory information from the facial nerve (Fig. 1o)^30^ and the nodose ganglia, which contains the cell bodies for afferent vagal neurons that innervate the esophagus and lower GI tract^24,31^. We also observed strong labelling in the caudal part of the spinal nucleus of the trigeminal nerve, which receives pain and temperature information from the oral cavity and face^32^. For all these structures, labeling was stronger on the side ipsilateral to the injection site (Fig. 1n–o) and was specific to inputs to CGRP neurons, as no GFP expression was observed after injection of PRV-Introvert-GFP into wild-type mice (Supplemental Fig. 2c). Thus, CGRP neurons receive prominent input from sensory pathways that innervate the oral cavity and esophagus as well as the GI tract, consistent with the observation that a subset of these neurons is rapidly activated during ingestion.

### Activation of CGRP neurons during licking is not potentiated by post-ingestive signals

If CGRP neurons drive satiation^3^, then their activity would be predicted to increase as the meal progresses. One way this could happen is that post-ingestive signals potentiate CGRP neuron responses to licks, such that this time-locked activation increases in magnitude as the meal proceeds (Supplemental Fig. 1d). However, we observed minimal evidence for this during self-paced ingestion. For example, lick responses for Ensure during intermittent access tests were similar in magnitude at the beginning and end of the 60 min session (e.g. 0.015 ± 0.003 vs. 0.013 ± 0.004 mean z-score per lick, *P* = 0.56, Supplemental Fig. 1e), despite the fact that mice consumed a large volume of Ensure that would be predicted to promote satiation (5237 ± 537 licks, n = 4 mice).

**Figure 2,:**
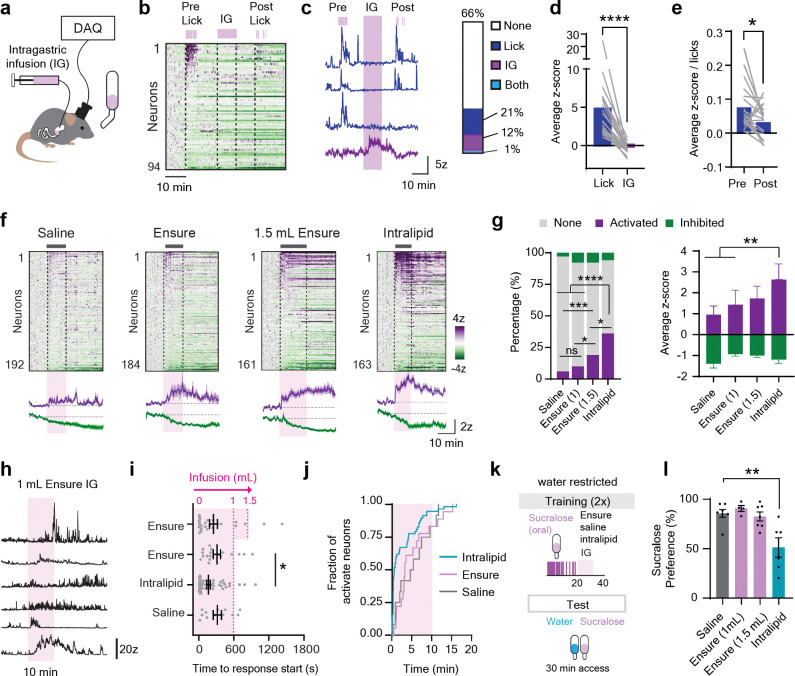
CGRP neurons show limited responses to post-ingestive nutrients. **a**, Mice licked for Ensure before and after a 1 mL intragastric infusions of Ensure during single-cell calcium imaging of CGRP neurons. **b**, Heatmap showing neural responses to either licking or IG infusion. **c**, Example traces and quantification of neural responses. **d**, Average z-score during either licking or IG infusion for ingestion-activated neurons. **e**, Mean lick-normalized responses to Ensure pre vs. post IG infusion. **f**, Heatmaps of neural responses to 1 or 1.5 mL IG infusions of saline, Ensure, or intralipid. **g**, Left, Quantification and right, mean z-scored responses to IG infusion of activated and inhibited neurons. **h**, Example neural traces in response to a 1 mL Ensure IG infusion. **i**, Time to the response onset relative to the start of the start of the IG infusion. **j**, Fraction of activated neurons activated over 1 mL IG infusions. **k**, Paradigm for conditioned taste avoidance where sucralose is paired with an IG infusion of different solutions. **l**, Decreased preference for sucralose in a two-bottle choice test after pairings with IG intralipid, but not other solutions. **** P < .0001, *** P < .001, ** P < .01. * P < .05.

To test this idea more rigorously, we equipped mice with intragastric (IG) catheters^33,34^ so that we could infuse nutrients directly into the stomach while recording CGRP neuron dynamics by microendoscopy (Fig. 2a). We then measured how lick responses were altered by IG infusion of nutrients. To do this, hungry mice were first given brief access (5 min) to Ensure for oral consumption; the lick bottle was then removed and the mice were given an IG infusion of Ensure (1 mL over 10 min); the lick bottle was then returned and mice were given a second brief access period (5 min) for Ensure oral consumption (Fig. 2b–d).

We found that mice consumed more Ensure in the first access period than in the second (1096 ± 83 vs. 478 ± 111 licks, session 1 vs. 2, *P* = 0.002), and this was due to a decrease in the number of lick bouts initiated (12 ± 2 vs. 7 ± 2 bouts, session 1 vs. 2, *P* = 0.03). This confirms that the IG infusion was sufficient to promote satiation. However, the activation of CGRP neurons during licking was actually *reduced*, rather than increased, following IG nutrient infusion. This was due to both a decrease in the number of Ensure-responsive neurons (21% vs. 14%) and a decrease in the activation of those neurons per lick (0.07 ± 0.01 vs 0.03 ± 0.01 mean z-score per lick, *P* = 0.01, Fig. 2e). Thus, post-ingestive nutrients do not potentiate the rapid activation of CGRP neurons during licking, as might be expected if this activation functions to promote satiation.

### Post-ingestive signals only weakly modulate the tonic activity of CGRP neurons

An alternative possibility is that post-ingestive feedback activates CGRP neurons by increasing the tonic or “baseline” activity of these cells, which then functions to inhibit food intake. This tonic activation would be detected, during self-paced ingestion, as an increase in the baseline activity of CGRP neurons between bouts of licking (Supplemental Fig. 1f). However, we observed little evidence for this. Among the CGRP neurons that were activated by licking, we actually observed a *decrease* in their interbout activity as the session progressed, and this was greater for the caloric compared to the non-caloric solutions (Caloric 0.2 ± 0.2 vs non-caloric 1.4 ± 0.5 z-score, *P* = 0.03, Supplemental Fig. 1g,h). On the other hand, among the CGRP neurons that did not respond to licking, there was no change in activity as the meal proceeded (Supplemental Fig. 1i). We also looked at the interbout activity during the last 10 minutes of self-paced drinking sessions, regardless of the solution being consumed, and binned them based on the total volume consumed during that session. CGRP baseline activity did not scale with volume consumed (Supplemental Fig. 1j). During intermittent access tests, we only detected one CGRP neuron (out of 122 cells) that appeared to show ramping activation during ingestion (shown in Fig. 1e). Thus, the baseline activity of most CGRP neurons does not track total food consumed, suggesting that their activity is not driven by post-ingestive signals during a normal meal.

These findings are contrary to the conventional wisdom that CGRP neurons track GI signals, such as stomach distension, to trigger meal termination^1,3,35^. We therefore tested the role of GI signals directly by measuring responses of CGRP neurons to infusions of different solutions into the stomach.

We first recorded neural responses to IG infusion of Ensure, which we delivered in an amount (0.93 kcal; 0.1 mL/min over 10 min) that approximates a moderately-sized meal and robustly modulates many neural cell types that control food intake^13,14,24,33,36^. We found that only a small percentage of CGRP neurons (10%) were activated by Ensure IG infusion (Fig. 2f–g), and, importantly, the magnitude of this activation was not different from the response to equivolemic saline infusion (10% vs. 6% activated neurons, Ensure versus saline, *P* = 1), indicating that it does not reflect GI nutrient sensing. The neurons that were activated by Ensure infusion showed a heterogeneous mixture of phasic and tonic responses (Fig. 2h), most of which begin during the IG infusion (average latency to onset 5.2 ± 1.0 min, Fig. 2i–j). These IG-activated CGRP neurons were also distinct from the CGRP neurons that responded to licking (Fig. 2b–d). As a control, we confirmed that many CGRP neurons in these animals were activated by IP injection of CCK or the GLP-1 receptor agonist Exendin-4, indicating these neurons can respond to GI signals when administered at supraphysiologic levels (Supplemental Fig. 3a–d). However, few CGRP neurons appear to track distension caused by a moderately sized meal, consistent with our results from self-paced feeding.

It has been proposed that CGRP neurons are preferentially recruited following excessive food intake^1,35^. Consistent with this, we found that increasing the infusion to 1.5 mL of Ensure (1.5 kcal) increased the percentage of CGRP neurons responding (18%, Fig. 2f–g). We also tested whether a hypercaloric, pure fat stimulus would engage CGRP neurons. Indeed, IG administration of Intralipid (1 mL of 20%; 2 kcal) activated a larger percentage of CGRP neurons (36% vs 10%, Intralipid vs. 1 mL Ensure, *P* < .0001, Fig 2g), to a greater degree than either IG saline or Ensure (Ensure: 1.4 ± 0.4, Intralipid: 2.6 ±0.3 *z*, *P* < .009). Of note, while the overall response of CGRP neurons was biased toward activation, there was also a subpopulation of CGRP neurons that were inhibited (Fig. 2g).

We wondered why CGRP neurons showed a markedly stronger response to IG Intralipid compared to Ensure. One possibility is that the Intralipid infusion caused greater gastric distension, because ingested fat empties from the stomach slower than sugar^37^. To test this, we gave mice an IG infusion of either saline, Ensure or Intralipid (1.0 mL over 10 min) and then measured the contents in different parts of the GI tract at the end of the infusion (Supplemental Fig. 4a–c). As expected, Ensure and Intralipid emptied from the stomach slower than saline (Supplemental Fig. 4d), and, correspondingly, the stomach volume and the weight of the remaining stomach contents were greater for these solutions compared to saline (Supplemental Fig. 4b,e). However, there was no difference in any of these parameters between Ensure and intralipid. There was also no difference in the amount of Ensure versus Intralipid that reached the duodenum or jejunum during the experiment (Supplemental Fig. 4b). Thus, differences in gastric emptying or distension are unlikely to explain the differential response of CGRP neurons to sugar and fat.

Given that CGRP neurons are activated by aversive stimuli^1,4,8,11,12^, an alternative possibility is that IG infusion of fat, but not sugar, leads in some way to discomfort or malaise. To test this, we measured whether IG infusion of Intralipid (1.0 mL), Ensure (1.0 or 1.5 mL), or saline (1.0 mL) was sufficient to induce conditioned taste avoidance (CTA). Mice were trained by pairing, on two separate days, a novel tastant (flavored sucralose) with subsequent IG infusion (Fig. 2k). Mice then underwent a two-bottle preference test for sucralose and water (Fig. 2l). This revealed that IG infusion of Intralipid, but not Ensure, reduced the preference for paired sucralose (85.7% ±4.0 vs, 51.3 ±9.9% preference, IG saline vs. intralipid, *P* = 0.002). Thus, the presence of concentrated fat, but not Ensure or saline, in the GI tract appears to be aversive and activates CGRP neurons (Fig. 2f–g).

### Different populations of CGRP neurons respond to ingestion and visceral malaise

**Figure 3,:**
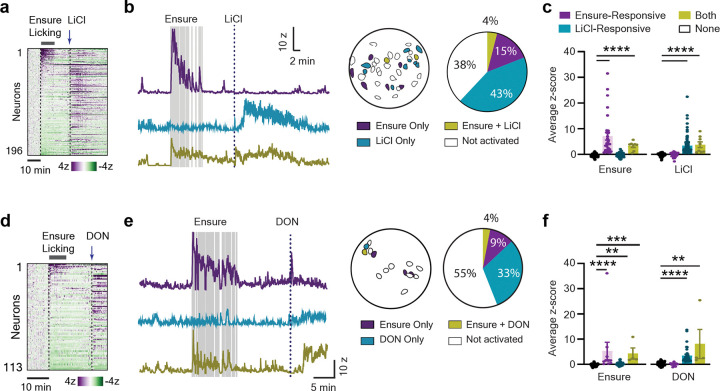
Distinct CGRP neurons respond to ingestion and malaise. **a**, Heatmap of neural responses to self-paced licking for Ensure and subsequent LiCl administration in the same recording session. **b**, Left, example responses of neurons that responded to Ensure, LiCl, or both stimuli. Vertical gray bars are licks. Right, spatial distribution of cells with each response type in one animal, and quantification of the percentage of each type of response across all mice. **c**, Quantification of the average z-score during different stimuli, for each category of neuron. **d**, Heatmap of neural responses for ensure and DON and **e**, Example traces and quantification. **f**, Mean z-score response of neurons based on the stimuli they respond to. **** P < .0001, *** P < .001, ** P < .01.

The discovery that a subset of CGRP neurons is rapidly activated by appetitive signals associated with ingestion (Fig. 1) raises the question of whether these are the same cells that respond to aversive signals associated with malaise. To test this, we gave fasted mice access to Ensure (10 min) followed by an i.p. injection of LiCl (84 mg/kg), which is commonly used to induce visceral malaise^5,10,38^ and activates CGRP neurons^1,9^. We found that a large subpopulation of CGRP neurons were activated by LiCl (43%, 3.4 ± 0.4 z-score, Fig. 3 a–c) and this was largely distinct from the neurons that were activated by Ensure (only 4% of neurons responding to both stimuli). These two subsets of cells were intermingled at the level of our 0.5 mm GRIN lens recordings (Fig. 3b). To confirm that aversive and appetitive signals activate different CGRP neurons, we repeated this experiment using Ensure followed by injection of “vomitoxin” (deoxynivalenol, or DON), a mycotoxin that causes gastrointestinal distress and nausea (Fig. 3d–f)^39^. We found that 33% of CGRP neurons were activated by DON (3.8 ± 0.53 average z-score), and these DON activated neurons were mostly non-overlapping with the cells that responded to Ensure (only 4% responding to both). Thus, aversive and ingestive signals activate different subsets of CGRP neurons in the PBN.

### The activation of CGRP neurons during ingestion is necessary for taste learning but not satiation

**Figure 4,:**
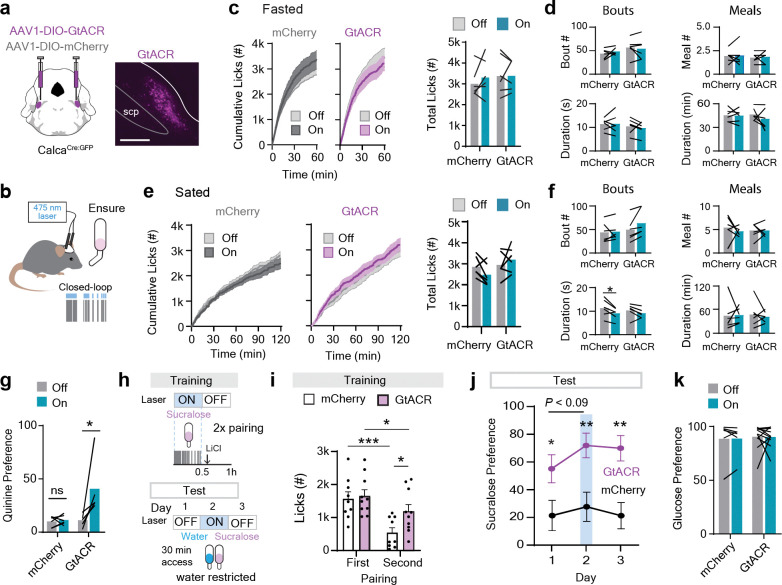
Rapid ingestion responses are important for learning but not satiation. **a**, *Calca*^Cre^ mice were injected with AAV1-DIO-GtACR or AAV1-DIO-mCherry and implanted with optic fibers. Right, image of GtACR expression in the PBN, scale bar 200 um. **b**, Schematic of mice receiving lick-triggered closed-loop optogenetic inhibition while given access to Ensure. Vertical gray bars are licks. **c**, There were no differences in total number of licks for Enusre in fasted mice. **d**, There were no changes in bout or meal number and duration in fasted mice. **e**, There were no differences in cumulative rate or total lick number in sated mice given Ensure access. **f**, Bout and meal number and duration were not different between groups. **g**, Continuous GtACR-inhibition of CGRP neurons increased preference for a bitter solution vs. water. **h**, Training and testing paradigm for conditioned taste avoidance, where CGRP neurons were selectively inhibited when mice were given access to sucralose during training. **i**, GtACR mice drank more sucralose than control mCherry mice on the second day of training. **j**, GtACR mice showed greater sucralose preference in two-bottle choice tests across consecutive days, where CGRP neurons were inhibited on day 2. **k**, CGRP inhibition had no effect on sweet preference in a two-bottle choice test. * P < .05, **P < .01, ***P < .001. scp, superior cerebellar peduncle.

We next sought to determine the function of the ingestion-triggered activation of CGRP neurons. To do this, we targeted the inhibitory opsin GtACR (or mCherry for controls) to CGRP neurons and implanted optical fibers bilaterally over the PBN for silencing (Fig. 4a). We first performed closed-loop (lick-triggered) silencing of CGRP neurons while mice consumed Ensure, to test whether the spikes in CGRP neuron activity during licking regulate food intake (Fig. 4b). Surprisingly, this manipulation had no effect on the amount of food consumed in either fasted or fed mice (Fig. 4c–f). Because CGRP neurons have been proposed to specifically regulate meal size^3^, we also searched for an effect of silencing on the temporal structure of food intake. However, there was no effect of silencing on the dynamics of cumulative food consumed (Fig. 4c,e), on bout size or number (Fig. 4d,f), or meal size or number (Fig .4d,f). We also performed continuous optogenetic silencing during self-paced Ensure ingestion to test for a role of tonic CGRP neuron activity, but this again had no effect on any aspect of feeding behavior in either fasted or fed mice (Supplemental Fig. 5a–d). As a positive control, we confirmed that silencing CGRP neurons in the same animals was sufficient to increase consumption of a bitter quinine solution (Fig. 4g), as previously reported^8,40^, confirming the functionality of the optogenetic implants. Taken together, these data indicate that CGRP neurons are not required for either satiation or the control of any aspect of meal microstructure during consumption of a liquid diet.

We next considered the possibility that the ingestion-triggered activation of CGRP neurons could be involved in learning. In this regard, CGRP neurons are known to be critical for CTA^5,10^, the process by which animals learn to associate a taste (the conditioned stimulus or CS) with an aversive signal such as LiCl (the unconditioned stimulus or US). CGRP neurons are activated by aversive stimuli such as LiCl^1,9^ (Fig. 3a–c) and their artificial activation can substitute for LiCl in driving CTA^5,10^, suggesting that CGRP neurons supply the US during aversive learning^38^. On the other hand, the CS (the taste) is thought to be encoded by parallel and downstream brain regions^9,38,41^. However, the discovery that a subset of CGRP neurons is activated by appetitive tastants led us to hypothesize that CGRP neurons might also supply information about the CS during learning.

To test this, we asked whether silencing CGRP neurons only during exposure to the CS is sufficient to block aversive learning. Mice were trained in a paradigm for CTA in which they first consume a novel tastant (sucralose, 30 min) and then receive an injection of LiCl, repeated across two training sessions (Fig. 4h). Remarkably, we found that inhibiting CGRP neurons only during the sucralose consumption was sufficient to block the development of CTA, as reflected by increased consumption of sucralose on day 2 of training (Fig.4i) and increased preference for sucralose in a subsequent two-bottle choice test (Fig. 4j, day 1). Inhibition of CGRP neurons during a subsequent two-bottle choice test further increased preference for sucralose, in agreement with the role of CGRP neurons in the retrieval of a CTA memory (Fig. 4j, day 2)^10^. Importantly, CGRP neuron inhibition did not interfere with the valence of sweet taste itself, as there was no difference in sucralose intake on the first day of training (1567 ± 210 vs. 1658 ± 186 licks, control vs. GtACR, *P* = 0.74; Fig. 4i) nor did it have an effect on preference for a sweet solution in animals that did not undergo CTA training (Fig. 4k)^40^. Together with our imaging results (Fig. 3), these findings reveal that separate populations of CGRP neurons track the CS and US during aversive learning, and that making this association requires CGRP neuron activity not only during exposure to the aversive US but also surprisingly during the appetitive CS (Fig. 1).

## Discussion

Satiation and sickness are closely connected interoceptive states. Overeating can quickly transform satiation into discomfort, and nausea is one of the major side effects of appetite-curbing drugs used to treat obesity. Thus, understanding how these two states are differentially represented in the brain is a longstanding and important question in physiology.

Parabrachial CGRP neurons are unique in that they are broadly implicated in the control of nausea and sickness^1,5,10^ but are also thought to contribute to normal satiation^3,12^. To understand how these two functions are performed by the same cells, we reinvestigated the dynamics and function of CGRP neurons during both feeding and malaise. Contrary to current models^35^, we find that CGRP neurons do not track cumulative food intake, and their activity is not necessary for meal termination or any aspect of self-paced food consumption. However, a subset of CGRP neurons is rapidly activated during feeding in a manner time locked to ingestion. We show that these ingestion-activated cells are distinct from the neurons that respond to visceral malaise, and their activation during CS consumption is required for animals to learn to associate a CS with aversive post-ingestive stimuli. These findings reveal a role for different subsets of CGRP neurons across multiple stages of aversive learning.

### CGRP neurons are dispensable for normal satiation

While many lines of evidence implicate CGRP neurons in the control of sickness, malaise and danger, their role in meal termination rests primarily on two observations. First, it was shown that CGRP neuron activity gradually increases over approximately one hour during feeding, which roughly correlates with the onset of post-prandial satiety^12^. Second, it was shown that silencing of CGRP neurons with tetanus toxin increases meal size in mice that are hungry^3^. These observations have led to the idea that CGRP neurons track post-ingestive feedback from the GI tract to regulate the timing of meal termination^35^.

We were unable to find evidence to support this model. Regarding CGRP neuron dynamics, we found that a subset of CGRP neurons (~20%) were activated by ingestion, but this response was transient and time-locked to licking, suggesting it reflects pre-gastric signals such as taste or esophageal stretch^14–16^ or, possibly, the initial arrival of food to the stomach^42^. We observed few cells that showed a ramping activation over tens of minutes that would be indicative of post-ingestive feedback from the stomach or intestines. Consistently, relatively few CGRP neurons were activated by an IG infusion of nutrients into the stomach, except under conditions where the infusion induced CTA. These observations are more consistent with a role of CGRP in regulating aversive states than physiologic satiety.

To test for the necessity of CGRP neurons in regulating food intake, we optogenetically silenced these neurons and measured the effect on self-paced food consumption. This failed to reveal any effect of CGRP neurons silencing on the amount of food consumed, or meal microstructure, in fasted or fed animals. We cannot explain why our results differ from a previous studying using tetanus toxin to silence these neurons^3^, but it is conceivable that chronic loss of CGRP neurons results in circuit-level adaptations that are important for feeding behavior. It is also possible that there are conditions that we did not test under which CGRP neuron activity is more important for meal termination. However, our results clearly show that CGRP neurons do not play a critical role in regulating food consumption under common conditions used in the lab, which is more in line with the idea that satiation is associated with populations of neurons that do not trigger aversion or malaise^43^.

### CGRP neurons are required during both the CS and US in aversive learning

Extensive evidence implicates CGRP neurons in aversive learning. For example, it has been shown that CGRP neurons are activated by diverse stimuli associated with danger or sickness^1,5,8,12^, and we confirmed in our imaging preparation that a subset of CGRP neurons (30–45%) are strongly activated by emetics such as LiCl and DON (Fig. 3). CGRP neurons were also activated by IG intralipid under conditions that induced a mild CTA (Fig. 2)^44^. These observations, together with extensive data from functional manipulations^5,10^, are consistent with the conclusion that CGRP neurons encode the US in aversive post-ingestive learning. In this context, we were surprised to find that a separate population of CGRP neurons was activated by ingestion of appetitive solutions, which typically function as the CS in models of post-ingestive learning. Moreover, blocking this activation by silencing CGRP neurons only during CS consumption prevented the formation of a CTA (Fig. 4). This indicates that the sequential activation of two separate populations of CGRP neurons – one that tracks the CS and one that tracks the US – is required for forming a taste memory.

We do not know how CGRP neuron activation during CS consumption contributes to CTA, but our data argue against a few potential mechanisms. First, silencing CGRP neurons had no effect on consumption of the CS during the first day of training, indicating that this learning defect is not indirectly due to a change in behavior. Second, we found that CGRP neurons were reliably activated by ingestion of familiar liquid diets that the mice had consumed many times, indicating that this activation does not encode primarily neophobia^12,45^ or novelty^9^. This is relevant because novel CS solutions tend to induce a stronger CTA due to the absence of latent inhibition^5,46,47^. Third, while it is possible that CGRP neurons encode some sensory features of the CS, our data suggest these neurons respond primarily to ingestion itself rather specific gustatory cues (Fig. 1).

An alternative possibility is that CGRP neuron activation during CS consumption acts as a permissive signal that is required, in some way, for subsequent creation of the taste memory in downstream circuits. In this regard, it has recently been shown that activation CGRP neurons by an aversive US is sufficient to reactivate the representation of the recently consumed taste in the downstream central amygdala (CeA)^9^. Thus it is possible that the activation of CGRP neurons during CS consumption is necessary to tag these downstream CeA neurons for subsequent reactivation. Testing these ideas will likely require identification of the molecular^48,49^ or other features^50^ that distinguish ingestion-activated CGRP neurons from other cells.

## Methods

### Animals

All procedures followed National Institutes of Health guidelines and were approved by the IACUC at the University of California, San Francisco. Mice were housed in a 12 h dark/light cycle (temperature and humidity controlled) *ad libitum* water and PicoLab 5053 chow unless otherwise noted. Heterozygous *Calca*^Cre^ (Jax # 033168) or C57Bl/6J mice were used for all experiments. Experimental animals were over 8 weeks old and included both males and females, which were randomly assigned to control or experimental groups for behavioral studies. Mice with intragastric catheters or used in microendoscopic imaging studies were singly housed, and all other mice were group-housed when possible.

### Intracranial surgeries

Mice were anesthetized with 2% isofluorane and placed on a Kopf small-animal stereotax. Viruses were pressure-injected into the parabrachial nucleus of *Calca*^Cre/+^ mice using glass capillaries and a Micro4 controller, at AP −5.1, ML ±1.5, DV −3.5 relative to bregma.

For tracing experiments, 0.15 μL pseudorabies-Introvert-GFP^17^ virus (NIH CNNV from the Herpesvirus Core at Princeton University) was injected unilaterally and mice were perfused 2–4 days later.

For imaging experiments, mice were injected with 0.1–0.2 μL of an AAV expressing cre-dependent GCaMP6s (AAV1-CAG-FLEX-GCaMP6s, Addgene 100842). During the same surgery, a commercially available GRIN lens from Inscopix (0.5 × 6.1 mm, 100–006647) was implanted at AP −5.0, ML 1.6, DV −3.6 or AP −5.2, ML 1.7, DV 3.7. Mice were baseplated 2–4 weeks after viral injection, and a subset of mice also received intragastric catheters as described below.

For optogenetic experiments, mice were bilaterally injected with either AAV1-hSyn-DIO-mCherry (Addgene 50459), or AA1-hSyn-DIO-GtACR2-FusionRed (Addgene 105677). Custom-made optogenetic fibers were implanted at AP −5.2, ML ±1.6, DV 3.6 and mice were given 2 weeks to recover before starting experiments. Animals with viral injections that missed on one or both sides of the brain were excluded.

### Intragastric catheterization surgery

Intragastric catheters were implanted as described^14,33^. Briefly, mice were anesthetized with isoflurane and sterile access catheters (C30PU-RGA1439, Instech Labs) were surgically implanted into the avascular stomach. The catheters were attached to sterile vascular access buttons (VABM1B/22, 22, Instech Labs) that were secured under the skin above the scapulae of the mouse, with an externalized port used to infuse solutions directly into stomach. An aluminum cap (VABM1C, Instech Labs) was placed over the port to protect it between experiments. Mice were given a minimum of one week to recover prior to the start of experiments.

### Histology

Mice were perfused with phosphate-buffered saline (PBS) and 30 mL of 10% formalin. Brains were post-fixed overnight then cryoprotected in 30% sucrose in PBS at 4°C, embedded in OCT, and stored frozen at −80C until processing. Brains were sectioned coronally at 30μm (every 3^rd^ section) on a cryostat. For determination of viral targeting, sections were imaged on a Zeiss LSM 510 confocal or Nikon Eclipse Ti2 widefield microscope.

For pseudorabies tracing, the pons, medulla, nodose and geniculate ganglia were collected. The ventral skull was transferred into PBS after overnight fixation and stored until ganglia dissection. All PRV-GFP tissue underwent immunohistochemistry for GFP. Briefly, tissue was blocked in 2% normal donkey serum/PBST (0.1% Triton-X) and incubated in primary antibody (1:2000 chicken anti-GFP, Abcam ab13970). Brains were left in primary overnight, whereas ganglia were incubated for 72 h. Tissue was then washed 3 times in PBS and incubated in secondary antibody at room temperature for 1–3 h (1:000 donkey anti-chicken Alexa Fluor-488A, Sigma-Aldrich SAB4600031). Tissue was washed an additional 3 times in PBS, mounted, and stored at 4°C.

We performed whole-slide imaging of brain tissue using a Nikon Eclipse Ti2 widefield microscope and NIS Elements with a custom JOBS program. Sections were imaged at 10x (8-bit) with consistent exposure and gain, and images were exported as PNG files for QUINT. Ganglia were whole-mounted and imaged using a Zeiss LSM 510 confocal.

### QUINT Analysis

Pseudorabies tracing analysis was done using QUINT (https://quint-workflow.readthedocs.io/en/latest/QUINTintro.html)^51^. To register sections to an atlas, images were downsized to 10% using *Nutil*, and LUT settings were uniformly adjusted in ImageJ to optimize feature visualization. Sections were registered to the Allen Brain common coordinate framework using *QuickNII* and *Visualign*. For automated cell detection, images were downsized to 50% of their original size and pixel and object classifiers were created in *Ilastik* to identify GFP-positive cells. *Ilastik* output files underwent a Glasbey transformation using ImageJ, and these output files and the final *Visualign* files were quantified in *Nutil* using object splitting. Data is reported as the area of each nucleus analyzed that had GFP-positive signal (GFP pixel area/region area sampled = load). Regions included were manually verified by visual inspection to guarantee model accuracy, and the top 10 hits across brains were included. The NTS was manually split at the obex, with sections containing the obex included in the caudal category. Regions with < 4000 pixels were filtered out, and the cerebellum and inferior and superior colliculi were excluded due to higher levels of background and damage. No cells were observed in these regions, although sparse labeling has been reported using monosynaptic rabies tracing^27^.

The *Calca*^Cre:GFP^ mouse line expresses nuclear-localized GFP that can be visualized with IHC. This endogenous GFP did not explain the expression pattern seen with PRV-GFP, as we did not see GFP in regions of interest in *Calca*^Cre:GFP^ mice without PRV-GFP injections (Supplemental Fig. 2c).

### Gastric emptying and distension

Fasted C57Bl/6J mice with IG catheters received a 1 mL infusion (saline, 20% intralipid, or Ensure) using a syringe pump (Harvard Apparatus, 70–2001) over 10 minutes (0.1 mL/min). Mice were perfused at the end of the infusion with 10 mL PBS and 10 mL formalin. During the perfusion, suture was used to ligate the pyloric sphincter. The GI tract was then dissected out, and additional sutures were placed 10 and 20 cm distal to the pyloric sphincter in the intestine. Infused solutions were not observed below 20 cm. GI segments were weighed before and after removal of contents. Content weight was normalized to body weight (g per 20 g body weight)^24^. Gastric emptying = (infused solution weight - stomach contents)/infused weight. Gross stomach area was measured from calibrated images in ImageJ.

### Pharmacology

All compounds were prepared in 0.9% sterile saline and stored at −20C prior to use, except for lithium chloride (LiCl), which was stored at 4°C. Compounds consisted of CCK octapeptide (10–30 mg/kg; Tocris 1166), Exendin-4 (30–150 mg/kg; Tocris 1933), LiCl (84–100 mg/kg), or deoxynivalenol (DON, 2.5 mg/kg; Tocris 3976). All solutions were administered at a volume of 10μL/g BW.

### Optogenetics

Mice were habituated to bilateral fiber optic cables (Doric Lenses) before tests. A 473 nm laser delivered 0.8–1 mW of blue light per fiber either continuously or closed-loop (triggered by licks; laser off after 2 s without licking). Intake was monitored using lickometers in a Coulborn Habitest Modular System, which also controlled the laser. Sessions were excluded if the lickometer failed, mice did not engage (< 100 licks or > 10 min latency to lick), or the patch cord uncoupled. For Ensure consumption experiments, mice fasted overnight or sated were given 1–2h access to Ensure as noted. For two-bottle choice tests, mice were water-deprived overnight and given access to water and either 0.5 mM quinine or 0.67 M glucose for 30 minutes, with and without continuous laser inhibition. Mice underwent two sessions for each experimental condition, and the order was counterbalanced across groups for the laser on and off condition. All solutions were familiar.

Meals were defined by an inter-meal interval (interval between licks) of at least 5 minutes with a minimum intake of 5 licks (equivalent to ~ 0.05 g Ensure). Bouts were separated by an inter-lick interval of 5 seconds and were at least 3 licks long. Data points for each animal are an average of 1–3 replicate sessions. For cumulative intake of Ensure, data was binned into 1 second intervals.

### Conditioned taste avoidance

Water restricted mice underwent 4 days with 30-minute one-bottle training sessions each day, followed by 3 days of 30-minute two-bottle choice tests. Training sessions alternated between access to water and sucralose (0.8 mg/ml sucralose in saline). Sucralose was paired with continuous laser inhibition followed by 100 mg/kg IP LiCl with no laser inhibition, whereas water days had no laser or injection. Two bottle- choice tests were between water and sucralose, and laser inhibition only occurred on day 2 of the two-bottle tests. Mice also received daily 2 h water access in their home cage in the afternoon to provide additional hydration throughout the 7d experiment.

### CTA to IG infusions

Methods were similar as described above, although wild-type mice were given sucralose access paired with subsequent IG infusions (1 mL intralipid, Ensure, or saline, and 1.5 mL Ensure at a rate of 0.1 mL/min) instead of LiCl injections, and there was no laser. Only one day of the two-bottle choice test was performed. Some mice went through two rounds of training with different IG infusion pairings, where in the second test the sucralose was flavored with orange kool-aid.

### Microendoscopic imaging

#### Data Acquisition

Microendoscopic videos were acquired using Inscopix acquisition software (v151) at 20Hz, 8 gain, 0.4–0.8 mm^−2^ 455 nm LED power, and 2x spatial downsampling. Data was recorded using nVista (v.3.0), nVoke (v2.0) or nVue (1.0, 2.0) systems. Prior to recording, mice were connected to cameras with the LED on for 10 minutes for habituation and stabilization of GCaMP6s signal. A baseline of 10 minutes was collected prior to all manipulations. In some cases, mice were head-fixed on custom-made treadmills to stabilize the recordings.

For self-paced licking experiments, mice were fasted overnight and placed in Coulborn chambers, and licking for various solutions (saline, 0.24 g/mL glucose, 20% intralipid, 0.8 mg/mL sucralose in saline, and Ensure) was tracked using a custom made capacitive lickometer connected to the Inscopix acquisition system. Intermittent Ensure access experiments were performed in a Davis Rig (MED-DAV-160M, Med Associates). Mice underwent three days of training and habituation prior to experiments^13^. For recordings, fasted mice were given repeating 60 s access to Ensure, with 120 s breaks between access trials, for a total of 15 trials over the course of 60 minutes. For IG infusions, mice were fasted overnight and received 1 or 1.5 mL delivery of Ensure, 20% Intralipid, or saline. Hormones were administered IP in fasted mice. For experiments comparing responses to Ensure and aversive agents, mice were given Ensure access for 10 minutes and then received either 84 mg/kg IP LiCl or 2.5 mg/kg DON after a 10-minute break. LiCl was paired with vanilla Ensure, and DON was paired with chocolate Ensure.

### Video processing

Videos were processed using Inscopix Data-Processing Software (v1.9.4). Videos were spatially (factor 5) and temporally (factor 2) binned to 4 Hz, spatially band-pass filtered, and motion corrected. Videos with uncorrectable motion were discarded. In some cases, neurons within the videos had two distinct motion patterns, and two motion-corrected versions of the videos were created using ROIs around specific subsets of neurons. Large motion artifacts were excised. Activity traces for individual neurons were extracted using the constrained non-negative matrix factorization (CNMF-E) pipeline built into the Inscopix software, with output units of change in fluorescence over noise. Neurons were manually refined to remove duplicates, motion artifacts, and over-segmentation. Neural traces from 4–8 mice per experiment were pooled.

### Data Analysis

Neural traces were z-scored to the 10-minute baseline prior to stimulus onset. Mean activity was then averaged over the first 5 and the first 10 minutes of solution access to capture differences in distribution of licking over time. Neurons with mean *z* > +1 in either epoch were classified as activated, and those with *z* < −1 were inhibited. Neural responses across the first 10 minutes were graphed and used to compare responses between solutions.

Bout activity was the mean z across all bouts during the 30-min session, normalized to lick count where indicated. Interbout activity was calculated from the same traces after masking out bouts; in some analyses it was further split into the first and last 10 min of the session. Bouts were defined in the same way as in behavioral assays.

For intermittent access experiments, activity was averaged over each 60-s access period. Neurons were categorized as activated if mean z ≥ +1 in more than 50% of trials. The consistency activated neural responses were the percentage of trials where the mean response was ≥ 1 *z*. “Start” and “end” values are the average z during the first and last three trials containing ≥ 3 licks, normalized to lick count.

To analyze single-cell responses to licking, neuronal traces were z-scored to the 10-s baseline immediately preceding the first lick in each bout, and responses were quantified over the subsequent 0–20 s. For bout-offset analysis, traces were re-z-scored to the 10 s preceding the final lick in a bout and quantified over the next 0–20 second. Only bouts ≥ 10 s in duration and separated by ≥ 10 s were analyzed to guarantee independent baselines. All qualifying bouts from a given neuron were averaged to yield one PSTH per cell. PSTHs were smoothed with a moving average window equal to one-third of the 0–20 s response window, and time to 50% of the peak (rise) or trough (decay) was determined.

Following IG infusions, neurons were classified as activated if they passed two criteria. First, that mean z ≥ +1 during the 10–15 min infusion or in the 10 min after the infusion. Second, that z-score was ≥ 1 more than 70% of the time during a sliding 60 second window. This captures sustained but not necessarily continuous responses and excludes brief transients. The first frame of that qualifying window was defined as the response start time.

For Ensure vs. aversive agents, Ensure responses were z-scored to the 10-min pre-access baseline. LiCl and DON responses were z-scored to the 5-min pre-injection baseline (to exclude any lingering Ensure effect) and scored with the same 60-s sliding window rule used for IG infusions.

Heatmaps were downsampled to 1 hz, and neural traces were smoothed by a moving average filter for 20 data points for graphing purposes only. Raw data were used for all quantitative analyses.

### Statistical Analysis

Data are shown as mean ± s.e.m. (error bars or shaded areas). For behavioral tests, sample size is the number of mice per group. For imaging, sample size is the number of neurons. Graphing and analyses were performed in GraphPad Prism 9 (GraphPad Software) or using custom scripts in MATLAB R2020a.

Normality was assessed with Shapiro-Wilk. For two-group comparisons, two-tailed Student’s t-tests were conducted with parametric data and Mann Whitney *U* tests with non-parametric data (paired or unpaired where appropriate). Air- versus Ensure-bout distributions (Fig. 1g) were compared with a Kolmogorov–Smirnov test. Lick count versus response magnitude was evaluated by simple linear regression. Comparisons across multiple groups were analyzed using one-way ANOVA (Holm-Šidák’s post hoc) or Kruskal Wallis (Dunn post hoc). Šidák’s multiple comparisons were used with two-way ANOVA.

Pairwise odds ratios for cell activation were obtained from 2×2 contingency tables for each condition pair (activated vs. not-activated) and applying Fisher’s exact test to estimate odds ratios with 95% confidence intervals. *P*-values were adjusted for multiple comparisons using a Bonferroni correction. Power calculations were used to predetermine sample size when prediction of effect size was possible. Experiments were not randomized and investigators were not blinded.

## Supplementary Material

Supplement 1
