# Supplementary material for: Ingestion-activated CGRP neurons control learning but not satiety
Source: bioRxiv. 2025 Oct 9:2025.10.08.681275. Preprint. [Version 1] doi: 10.1101/2025.10.08.681275 (PMC12632507; doi:10.1101/2025.10.08.681275)
Supplement: Supplement 1 [file NIHPP2025.10.08.681275v1-supplement-1.pdf]

bioRxiv preprint doi: https://doi.org/10.1101/2025.10.08.681275; this version posted October 9, 2025. The copyright holder for this preprint (which was not certified by peer review) is the author/funder, who has granted bioRxiv a license to display the preprint in perpetuity. It is made available under aCC-BY 4.0 International license.

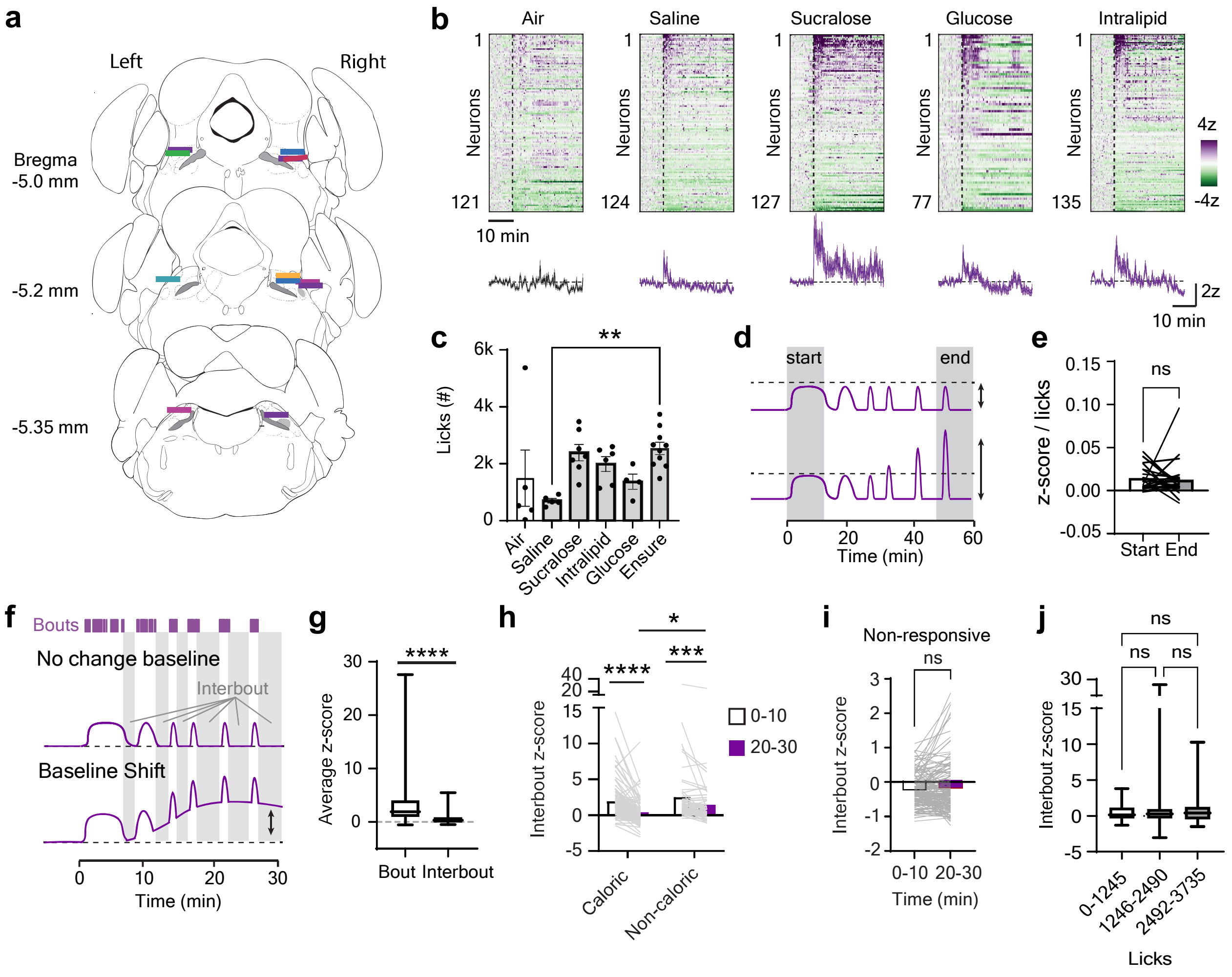

Supplemental Figure 1, Analysis of CGRP neuron responses to ingestion.
a, Approximate GRIN lens placements for all animals used for single-cell imaging. **b**, Heatmaps and averaged response of neurons to self-paced licking for various solutions. **c**, The amount mice licked for different solutions or air during single-cell imaging experiments. **d**, Model for how CGRP neurons responses to ingestion could change as over time as food consumption progresses. **e**, Mean z-scored responses normalized to number of licks for the first (start) and last (end) three trials of the intermittent access test. **f**, Model for looking at changes in tonic CGRP neuron activity (baseline shift) during food consumption. **g**, There were greater CGRP neural responses during licking bouts compared to interbout intervals for Ensure. **h**, Mean interbout z-score of activated neurons at the start and end of a licking session (first vs. last 10 minutes). **i**, Average interbout z-scored response in the first and last 10 minutes of Ensure access of neurons categorized as “non-responsive”. **j**, Interbout z-score of activated neurons in the last 10 minutes of a licking session for caloric solutions (Ensure, glucose, and intralipid), binned based on the total licks consumed by the end of the session. ns mean not significant. **** P < .0001, *** P < .001, ** P < .01. * P < .05

bioRxiv preprint doi: https://doi.org/10.1101/2025.10.08.681275; this version posted October 9, 2025. The copyright holder for this preprint (which was not certified by peer review) is the author/funder, who has granted bioRxiv a license to display the preprint in perpetuity. It is made available under aCC-BY 4.0 International license.

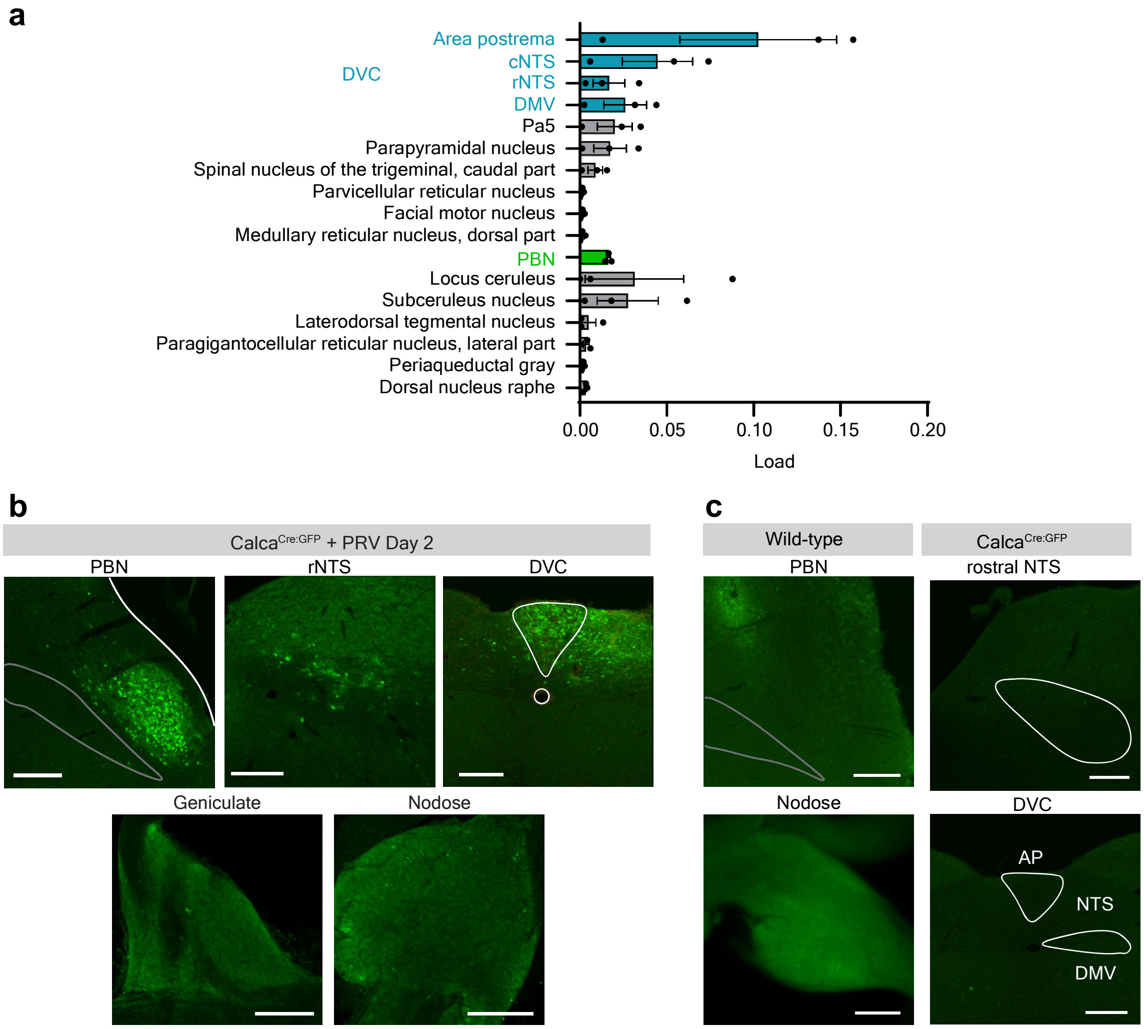

Supplemental Figure 2, Characterizationof PRV-GFP and brainstem inputs to CGRP neurons.
a, Quantification of the expression of pseudorabies virus-GFP on day 3 following injection into CGRP neurons in the parabrachial nucleus. Expression was calculated using QUINT, and are expressed as the number of pixels/area sampled (load) for three different mice. **b**, Example images of PRV-GFP on day 2 following injection, showing expression in the PBN and DVC, but not the geniculate or nodose ganglia. **c**, Controls showing that injection of PRV-GFP into wild-type mice does not lead to GFP expression in either the PBN or nodose ganglia, and endogenous expression of GFP in the Calca$^{Cre:GFP}$ mouse line is not present in the NTS and surrounding regions. scale bars, 200 um. AP, area postrema. NTS, nucleus of the solitary tract. DMV, dorsal motor vagus.

bioRxiv preprint doi: https://doi.org/10.1101/2025.10.08.681275; this version posted October 9, 2025. The copyright holder for this preprint (which was not certified by peer review) is the author/funder, who has granted bioRxiv a license to display the preprint in perpetuity. It is made available under aCC-BY 4.0 International license.

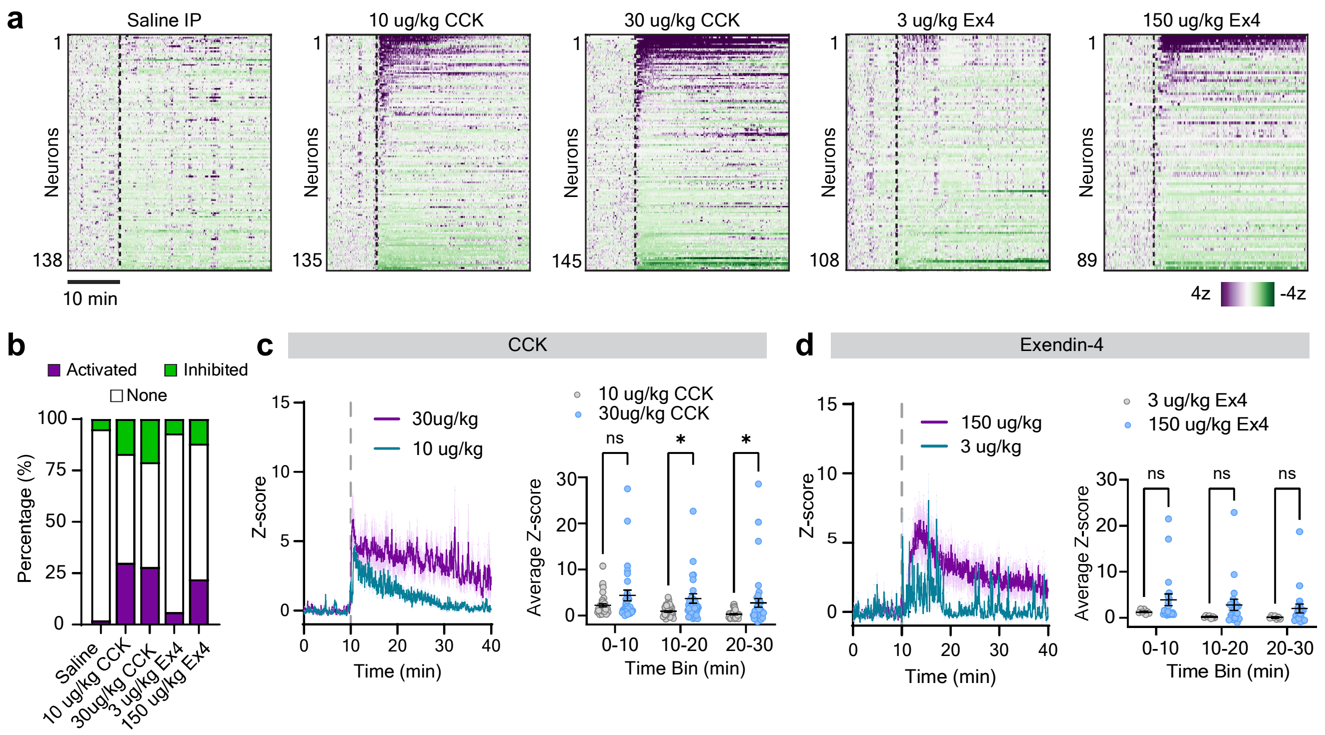

Supplemental Figure 3, Gut satiety hormones activate CGRP neurons.
a, Heatmaps of CGRP neural responses to i.p. injections of saline or different doses of CCK and the GLP-1 agonist, Exendin-4 (Ex4). **b**, Quantification of the neural responses following injections. **c**, Left, average response of the activated neurons to a low and high does of CCK. Right, quanitification of the mean z-scored response during different time bins following CCK injections showed that neurons had more sustained responses to 30ug/kg CCK, specifically during the 10-20 and 20-30 minute time bins. **d**, Left, Averaged trace showing response of activated neurons to two doses of Exendin-4. Right, there were no significant differences in responses over time between the Ex4 doses. ns mean not significant. * P < .05.

bioRxiv preprint doi: https://doi.org/10.1101/2025.10.08.681275; this version posted October 9, 2025. The copyright holder for this preprint (which was not certified by peer review) is the author/funder, who has granted bioRxiv a license to display the preprint in perpetuity. It is made available under aCC-BY 4.0 International license.

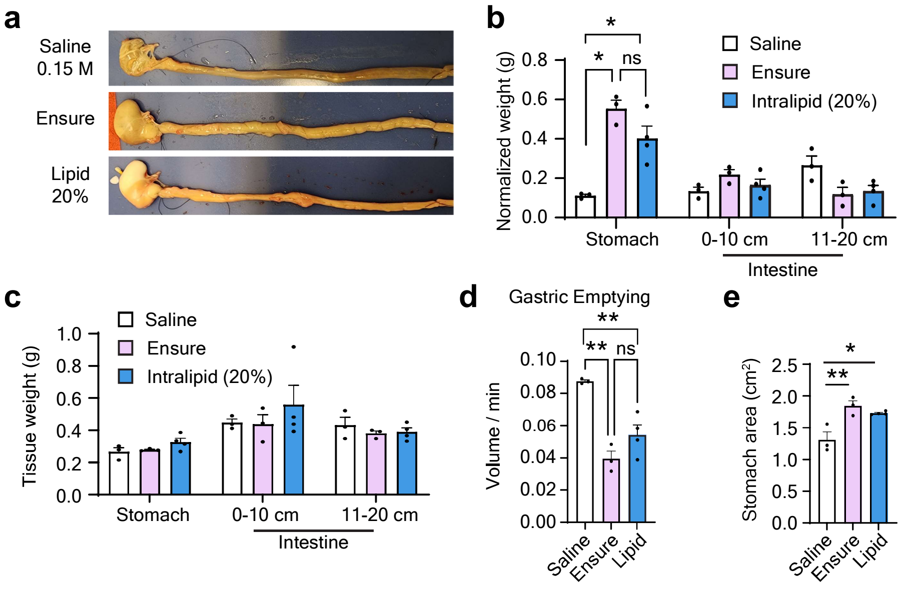

Supplemental Figure 4, Gastrointestinal changes following IG infusions.
a, Mice received a 1 mL intragastric infusions of saline, Ensure, or intralipid. At the end of the infusion, gastrointestinal tissue was collected and contents were analyzed. **b**, Weight of the contents of the stomach and different sections of the intestines. **c**, No differences in the weight of GI tissue (without contents) were observed between mice. **d**, Gastric emptying was significantly slower following infusion of Ensure or intralipid than saline. **e**, Surface area of the stomach was greater following infusion of Ensure and intralipid compared to saline. * $P < .05$, ** $P < .01$.

bioRxiv preprint doi: https://doi.org/10.1101/2025.10.08.681275; this version posted October 9, 2025. The copyright holder for this preprint (which was not certified by peer review) is the author/funder, who has granted bioRxiv a license to display the preprint in perpetuity. It is made available under aCC-BY 4.0 International license.

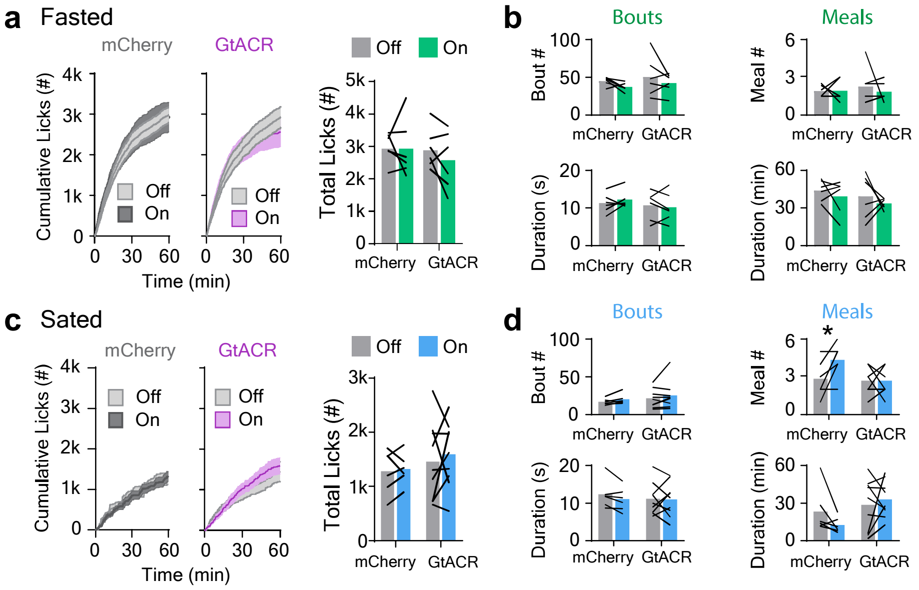

Supplemental Figure 5, Tonic inhibition of CGRP neurons does not impact food intake or meal microstructure.
Continuous bilateral inhibition of CGRP-PBN neurons expressing either mCherry or GtACR, during self-paced intake of Ensure. There was no effect on **a**, cumulative rate or total licks or **b**, bout and meal number and duration in mice that had been fasted overnight. There was also no effect on **c**, total licks or **d**, bout and meal number and duration in sated mice. * $P < .05$.
